# Evaluating the impact of deep learning‐based image denoising on low‐dose CT for lung cancer screening

**DOI:** 10.1002/acm2.70480

**Published:** 2026-01-24

**Authors:** Shih‐Sheng Chen, Hsiao‐Hua Liu, Ching‐Ching Yang

**Affiliations:** ^1^ Department of Medical Imaging Dalin Tzu‐Chi Hospital Chiayi Taiwan; ^2^ Department of Medical Imaging and Radiological Sciences Kaohsiung Medical University Kaohsiung Taiwan; ^3^ Department of Radiology Kaohsiung Veterans General Hospital Kaohsiung Taiwan; ^4^ Department of Medical Research Kaohsiung Medical University Hospital Kaohsiung Taiwan

**Keywords:** deep learning‐based denoising, low‐dose CT, lung nodule detection

## Abstract

**Purpose:**

Low‐dose CT (LDCT) is increasingly being adopted as a preferred method for lung cancer screening. However, the accompanying rise in image noise necessitates robust denoising strategies. Therefore, this study compared LDCT images with their denoised counterparts using objective image quality metrics and key nodule‐related features.

**Methods:**

The dataset utilized in this study was chest CT scans for lung cancer screening, sourced from the LDCT and Projection Data collection. Seven deep learning‐based image denoising methods were used in this work. The denoising performance was evaluated using root‐mean‐square error (RMSE), peak signal‐to‐noise ratio (PSNR), structural similarity index measure (SSIM), nodule size, CT density, and Lung‐RADS classification.

**Results:**

For solid nodules, denoising improved SSIM from 51% to 60%–64%, reduced RMSE from 137.13 HU to 62.40–78.30 HU, and increased PSNR from 23.91 dB to 28.59–30.51 dB. It also reduced the percent difference in diameter (PD_dia_) from 2.05% to 1.44%–1.52%, in volume (PD_vol_) from 5.95% to 4.43%–4.70%, in mean HU value (PD_HU_) from 24.40% to 8.54%–15.33%. For subsolid nodules, denoising improved SSIM from 47% to 57%–61%, reduced RMSE from 110.87 HU to 54.62–63.96 HU, and increased PSNR from 25.78 dB to 30.53–31.61 dB. Before denoising, the PD_dia_, PD_vol_ and PD_HU_ were 15.41%, 40.16% and 10.69%, respectively, which were 7.54%–15.94%, 17.54%–29.29%, and 6.10%–8.25% after denoising. These improvements led to higher Lung‐RADS categorization accuracy for solid nodules, while subsolid nodules remained more affected by noise and denoising‐induced bias.

**Conclusion:**

The integration of denoising techniques into LDCT workflows could potentially enhance early lung cancer detection without increasing radiation exposure. Nonetheless, validating their influence on diagnostic performance remains crucial for clinical adoption.

## 1 INTRODUCTION

Pulmonary nodules play a critical role in the early detection and diagnosis of lung cancer, as they are often the earliest radiographic manifestations of the disease.^1^ A solid nodule is defined as a focal opacity that completely obscures the underlying lung parenchyma within its boundaries, whereas a subsolid nodule, further classified as either a pure ground‐glass nodule or a part‐solid nodule, does not completely obscure the lung architecture.^2^ Solid nodules can represent a wide range of etiologies, including benign granulomas, focal scarring, intrapulmonary lymph nodes, primary lung malignancies, and metastatic lesions.^3^ In contrast, subsolid nodules carry a higher overall risk of malignancy compared with solid nodules. Transient subsolid nodules are commonly associated with inflammatory or infectious processes such as pneumonia or alveolar hemorrhage, while persistent subsolid nodules are of greater clinical concern, as they often correspond to lesions within the adenocarcinoma spectrum, ranging from adenocarcinoma in situ to minimally invasive adenocarcinoma and invasive adenocarcinoma.^4^ To guide clinical decision‐making and standardize management, follow‐up CT examinations are recommended for incidental pulmonary nodules detected on imaging.^5^, ^6^ The Fleischner Society guidelines provide size‐based recommendations for solid nodules, classifying them into three categories based on average diameter: <6 mm, 6–8 mm, and >8 mm. Nodules smaller than 6 mm typically require no further action, those between 6 and 8 mm warrant imaging follow‐up, and nodules larger than 8 mm usually require more aggressive diagnostic investigation. For subsolid nodules, the guidelines further refine management strategies: pure ground‐glass nodules and part‐solid nodules <6 mm generally do not require intervention, while ground‐glass nodules ≥6 mm and part‐solid nodules ≥6 mm necessitate follow‐up imaging and, depending on persistence or growth, more aggressive diagnostic workup. Given the frequency with which follow‐up imaging is performed in patients with pulmonary nodules, there is increasing emphasis on balancing diagnostic accuracy with radiation safety.^7^ Low‐dose CT (LDCT) techniques are especially important in this context, as they substantially reduce cumulative radiation exposure without compromising nodule detection.^8^, ^9^ This reduced radiation dose is especially important for screening purposes, where individuals may undergo repeated imaging over time. However, the accompanying rise in image noise necessitates robust denoising strategies. A wide range of techniques, from traditional filters and iterative reconstruction to cutting‐edge deep learning models, have been developed to mitigate noise while preserving diagnostic fidelity.^10^, ^11^, ^12^, ^13^ For such approaches to be clinically meaningful, they must not only improve image quality but also maintain consistent performance in tasks directly relevant to patient care. Accordingly, this study compared LDCT images with their denoised counterparts using objective image quality metrics and key nodule‐related features, with the goal of linking algorithmic improvements directly to diagnostic performance.

## 2 METHODS AND MATERIALS

### 2.1 Chest CT scans

The dataset utilized in this study was chest CT scans for lung cancer screening, sourced from the LDCT and Projection Data collection.^14^ The library consists of normal‐dose CT (NDCT), LDCT and clinical reports providing the location and diagnosis for positive findings, including snapshots of the identified findings delineated by radiologist‐drawn regions of interest. The patient scans were acquired using Siemens Healthineers scanners (SOMATOM Definition AS+ and SOMATOM Definition Flash) with a helical scan mode and without contrast enhancement. NDCT scan was performed at a tube potential of 120 kV, with rotation times of either 0.28 or 0.3 sec. Dose optimization was achieved through automatic exposure control (CareDose4D) with a quality reference of 70 mAs, resulting in a mean volume CT dose index (CTDI_vol_) of approximately 6.6 mGy. LDCT scans were generated using a validated noise‐insertion method applied to full‐dose projection data. The noise‐insertion technique accounted for various factors influencing image quality, including the bowtie filter effects, automatic exposure control, and electronic noise.^15^ The simulated low‐dose projection data corresponded to approximately 10% of the full‐dose level. CT scans were reconstructed using the FBP algorithm with a B50f reconstruction kernel, a slice thickness of 1.5 mm, and an image matrix resolution of 512 × 512 pixels. The field of view varied between 300 and 500 mm, depending on patient size.

### 2.2 Deep learning‐based denoising methods

Hyperparameter optimization is a critical and indispensable step in the process of fairly comparing different deep learning methods, as it ensures that each model is trained under its most favorable conditions, thereby providing a more reliable and unbiased evaluation of their true performance and effectiveness. Eulig et al. introduced a standardized benchmark framework designed to enable fair and consistent evaluation of various deep learning‐based LDCT denoising methods.^16^ In this study, seven deep learning‐based denoising methods were implemented following their publicly available benchmark implementations, ensuring consistency, comparability and reproducibility of results (https://github.com/eeulig/ldct‐benchmark). CNN10 (Convolutional Neural Network‐10) is one of the earliest convolutional neural network models applied to LDCT denoising, using a relatively shallow architecture to reduce noise while preserving anatomical structures, though it is limited in capturing complex image features.^17^ REDCNN (Residual Encoder‐Decoder Convolutional Neural Network) improved upon this by using an encoder‐decoder architecture with residual learning, enabling more effective noise suppression while preserving structural details.^18^ ResNet (Residual Neural Network) introduced deeper architectures with residual skip connections, improving gradient flow and achieving better performance in both quantitative metrics and visual quality.^19^ QAE (Quantized Autoencoder) is based on REDCNN but integrates quantization into training, making the model more hardware‐efficient and robust to deployment on low‐bit systems.^20^ WGANVGG combines a Wasserstein Generative Adversarial Network with perceptual loss derived from a pre‐trained Visual Geometry Group Network, enhancing texture realism and detail retention in denoised images.^21^ This method outperforms pixel‐wise loss models by preserving high‐frequency features and natural‐looking textures. DUGAN (Generative Adversarial Networks With Dual‐Domain U‐Net‐Based Discriminators) denoises in both spatial and gradient domains, effectively suppressing various types of noise and maintaining sharp edges, improving robustness across varying scan conditions.^22^ TransCT (Dual‐path Transformer for Low Dose Computed Tomography) leverages a transformer‐based architecture to capture long‐range dependencies, enabling superior structural preservation and noise reduction compared to convolutional approaches.^23^ Unlike CNNs, TransCT's attention mechanisms allow it to model global context, which helps retain spatial coherence in large anatomical structures. Table 1 summarized the LDCT denoising algorithms used in this study.

**TABLE 1:** LDCT denoising algorithms.

Model	Type/Architecture	Domain	Loss function	# Of parameters
CNN10	10‐layer CNN	Image	MSE	∼0.9 × 10^6^
REDCNN	Residual encoder‐decoder CNN	Image	MSE	∼1.4 × 10^6^
ResNet	Residual network	Image	MSE	∼1.2 × 10^6^
QAE	Quadratic autoencoder (quadratic convolutions)	Image	MSE	∼1.5 × 10^6^
WGANVGG	Wassertein GAN + perceptual VGG loss	Image	Adversarial + perceptual	∼5.2 × 10^6^
DUGAN	Dual‐domain U‐Net GAN	Image + gradient	Adversarial + MSE	∼12.3 × 10^6^
TransCT	Transformer‐based CNN	Image	MSE	∼24 × 10^6^

MSE: mean‐squared error.

### 2.3 Evaluation metrics

Root‐mean‐square error (RMSE) evaluates the average difference between pixel intensities in the test image and a reference image, with lower values indicating better fidelity. The mathematical expression of RMSE was shown as follows:

(1) $$\mathrm{RMSE}\;=\sqrt{\frac{\sum_{i=1}^{V}{\left(\mathrm{IM}G_{\mathrm{NDCT}}-\mathrm{IMG}\right)}^{2}}{V}}\;$$

where IMG_NDCT_ is NDCT image, and IMG is LDCT image either before or after denoising. V denotes the number of voxels within the whole image. Peak signal‐to‐noise ratio (PSNR) quantified the ratio between the maximum possible signal and the noise, where higher values suggest improved image clarity. The mathematical expression of PSNR was shown as follows:

(2) $$\mathrm{PSNR}\;=20\mathrm{lo}g_{10}\frac{HU_{\max}}{\mathrm{RMSE}}$$

where HU_max_ is the maximum Hounsfield Unit (HU) value of the image. Structural similarity index measure (SSIM) measures perceived image quality by comparing structural information, luminance, and contrast between the test and reference images; values closer to 1 indicate higher structural similarity. The mathematical expression of SSIM was shown as follows:

(3) $$\mathrm{SSIM}\;=\frac{\left(2\mu_{x}\mu_{y}+C_{1}\right)\left(2\sigma_{\mathrm{xy}}+C_{2}\right)}{\left(\mu_{x}^{2}+\mu_{y}^{2}+C_{1}\right)\left(\sigma_{x}^{2}+\sigma_{y}^{2}+C_{2}\right)}\;$$

where μ_x_ and σ_x_ are the mean and standard deviation (SD) of IMG_NDCT_, and μ_y_ and σ_y_ are the mean and SD of IMG. σ_xy_ denotes the covariance of IMG_NDCT_ and IMG. C_1_ and C_2_ are small constants to stabilize the division with weak denominator. RMSE, PSNR, and SSIM were computed for the entire reconstructed image with a matrix size of 512 × 512 (denoted as RMSE_global_, PSNR_global_, and SSIM_global_) and for localized subimages of 32 × 32 pixels that specifically encompassed lung nodules (denoted as RMSE_local_, PSNR_local_, and SSIM_local_), enabling a comprehensive evaluation of both overall image fidelity and localized feature preservation. Contrast‐to‐noise ratio (CNR) assesses the detectability of nodules by comparing the contrast between nodules and surrounding tissue relative to background noise, which is critical for ensuring that denoising preserves clinically relevant features. The mathematical expression of CNR was shown as follows:

(4) $$\mathrm{CNR}\;=\frac{\left|\mu_{\mathrm{nodule}}-\mu_{\mathrm{parenchyma}}\right|}{\sigma_{\mathrm{parenchyma}}}\;$$

where μ_nodule_ is the mean HU value of lung nodule, and μ_parenchyma_ and σ_parenchyma_ represent the mean HU value and SD of lung parenchyma, respectively. A CNR of 1.0 corresponds to a situation in which the image contrast between the nodule and normal lung tissue is equal to the statistical fluctuation of the normal lung tissue.

In addition to conventional objective image quality metrics, this study analyzed key nodule characteristics provided by AVIEW LCS (Coreline Soft, Seoul, Korea), a commercially available deep learning‐based computer‐aided detection (CAD) platform, to evaluate the clinical impact of deep learning‐based denoising methods in LDCT.^24^ For quantitative features, the percent difference (PD_feature_) between measurements obtained from NDCT and those from LDCT before and after denoising was calculated, where values approaching zero indicated greater similarity of lung nodule characteristics. The mathematical expression of PD_feature_ was shown as follows:

(5) $$\;PD_{\mathrm{feature}}=\frac{\left|m_{\mathrm{NDCT}}-\;m\right|}{m_{\mathrm{NDCT}}}\;\times 100\%$$

where m_NDCT_ represents a given nodule feature measured on NDCT, and m denotes the corresponding value obtained from LDCT either before or after denoising. Based on Equation (5), percent difference was calculated to compare nodule diameter, nodule volume and the mean HU value, which were denoted as PD_dia_, PD_vol_, and PD_HU_, respectively. Additionally, the CAD system classified the detected lung nodules into relevant categories according to the Lung Imaging Reporting and Data System (Lung‐RADS), a standardized classification system developed by the American College of Radiology to assess findings from LDCT lung cancer screening.^25^ It categorizes lung nodules based on their size, appearance, and growth into scores from 0 to 4X, which correspond to levels of suspicion for malignancy. Lower categories (Lung‐RADS 1 to 2) indicate benign or likely benign findings with routine annual screening recommended, while higher categories (Lung‐RADS 3 to 4X) suggest suspicious findings requiring shorter‐term follow‐up or additional diagnostic testing. The differences in nodule categories between NDCT and LDCT, before and after denoising, were assessed to determine the impact of image denoising on clinical interpretation.

## 3 RESULTS

**FIGURE 1:**
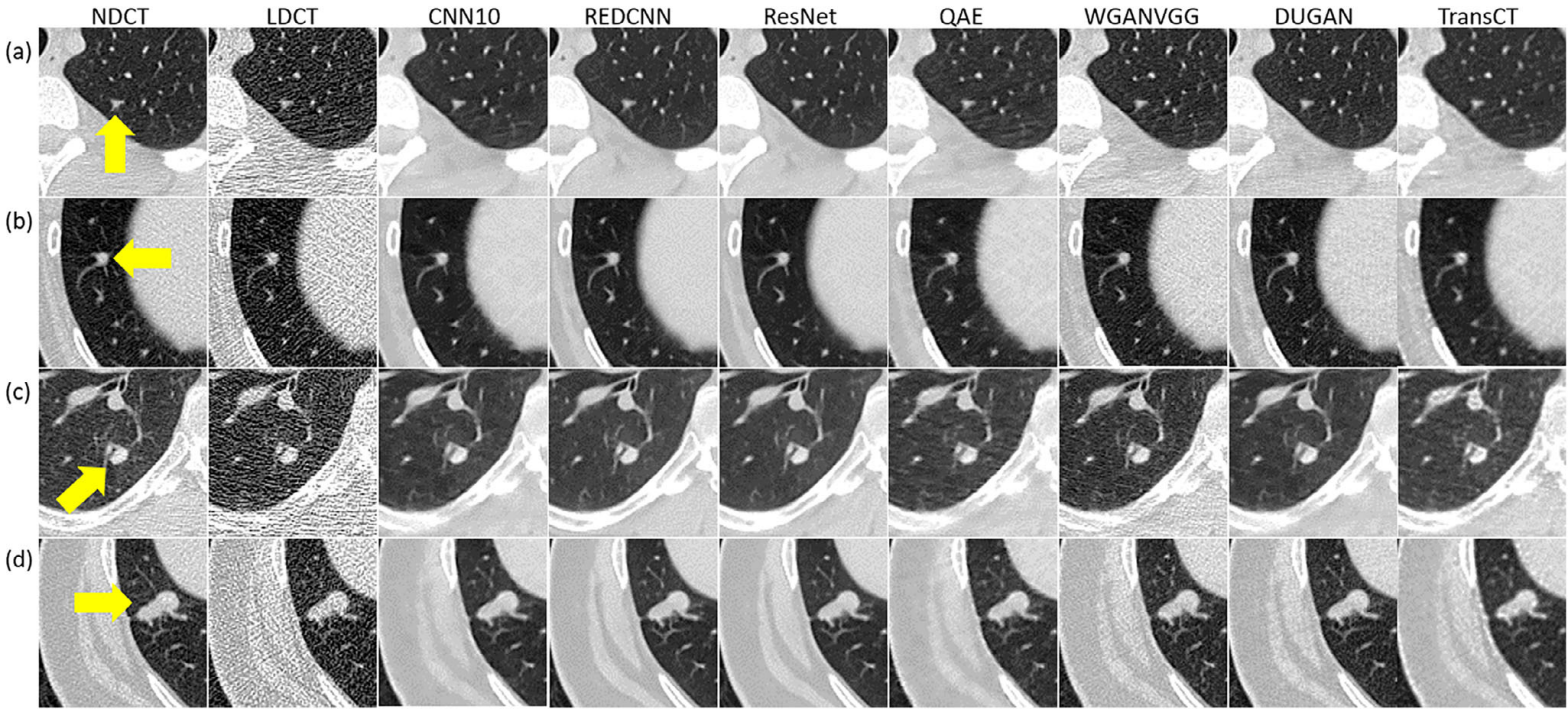
Axial images of solid nodules classified as Lung‐RADS (a) category 2, (b) category 3, (c) category 4A, and (d) category 4B on NDCT (window level/window width = ‐239/1568 HU).

**FIGURE 2:**
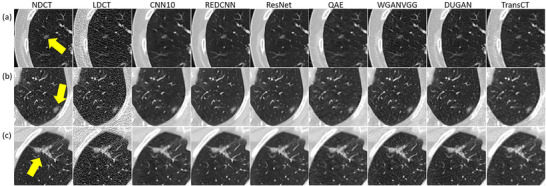
Axial images of subsolid nodules classified as Lung‐RADS (a) category 2, (b) category 3, and (c) category 4A on NDCT (window level/window width = ‐239/1568 HU).

**FIGURE 3:**
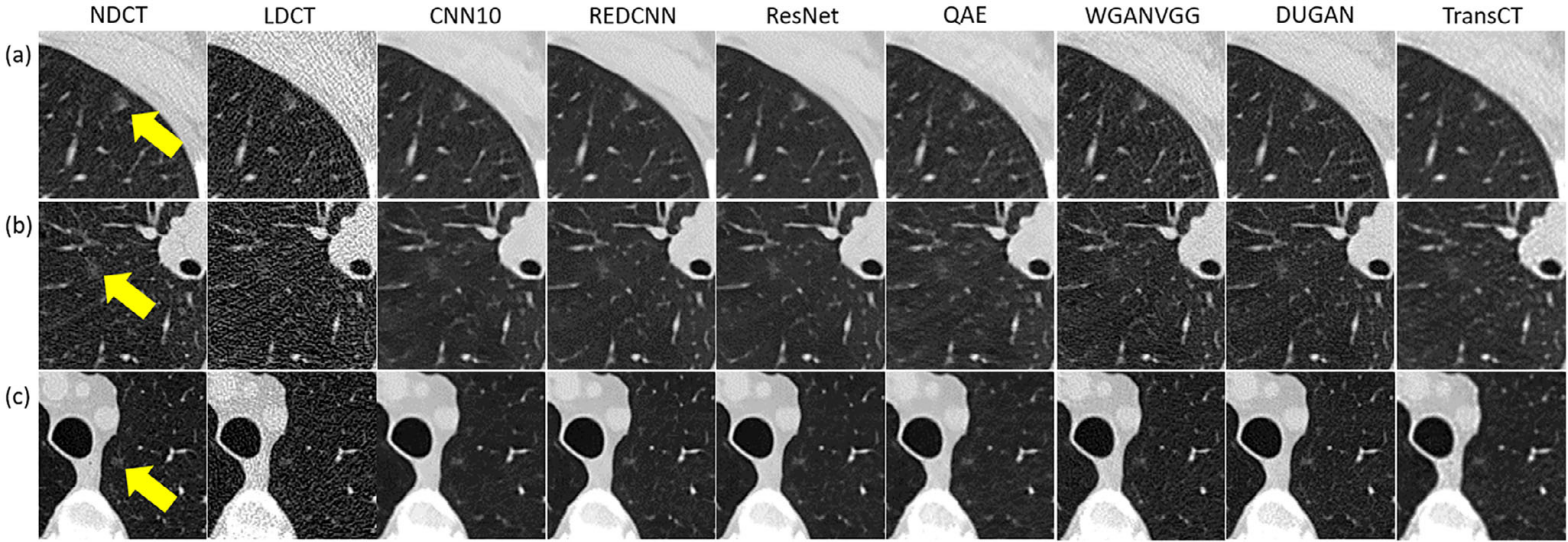
Axial images of subsolid nodules not detected by CAD on NDCT. (a) C095, detected on DUGAN; (b) C179‐7, detected on CNN10, REDCNN, ResNet,, DUGAN and TransCT; (c) C218‐4 (window level/window width = ‐239/1568 HU).

**FIGURE 4:**
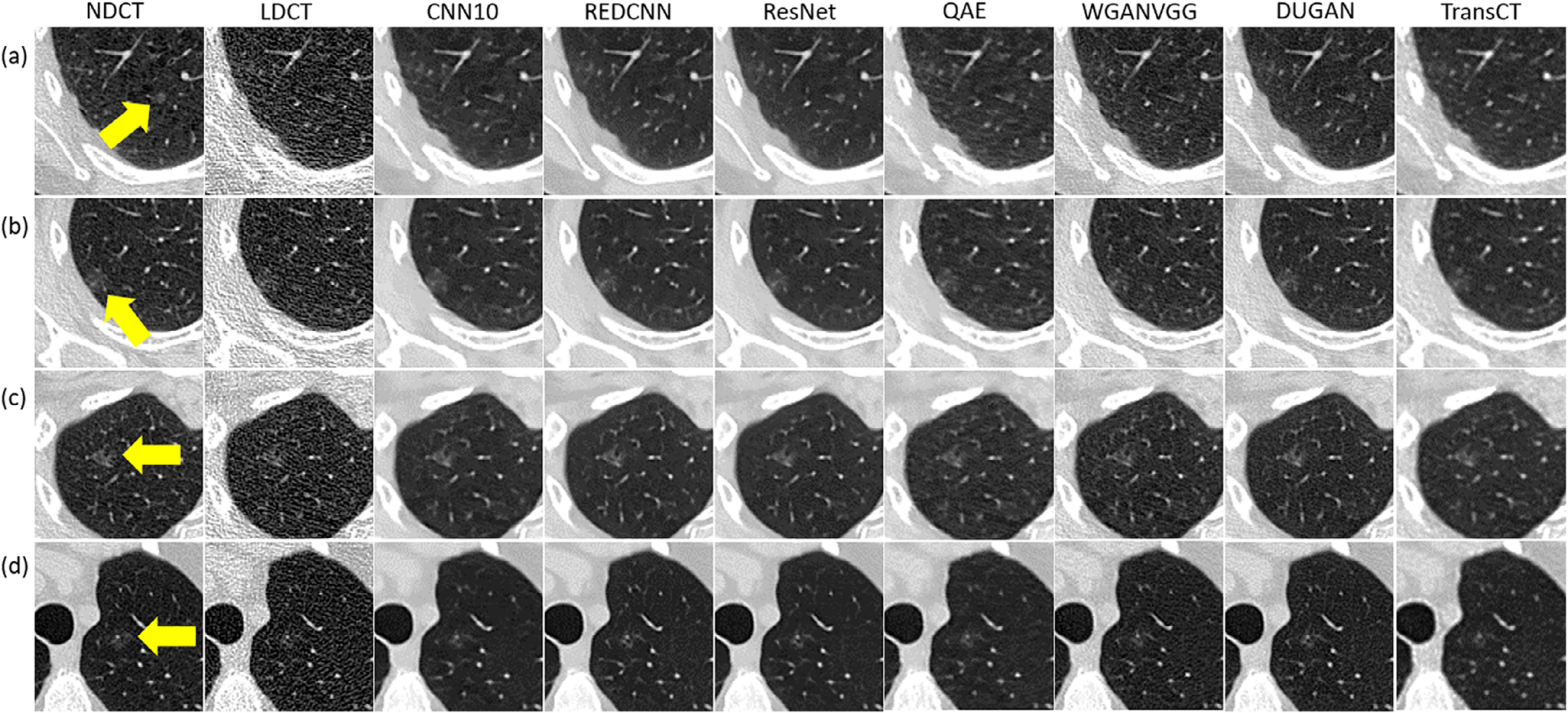
Axial images of subsolid nodules not detected by CAD on LDCT or denoised LDCT. (a) C081, detected on NDCT, REDCNN, ResNet, and DUGAN; (b) C179‐4, detected on NDCT and QAE; (c) C179‐5, detected on NDCT, LDCT, and TransCT; (d) C218‐1, detected on NDCT (window level/window width = ‐239/1568 HU).

Table 2 summarizes characteristics for patients with solid nodules, including age, CTDI_vol_ and Lung‐RADS classification for nodules in NDCT. Corresponding data for patients with subsolid nodules are summarized in Table 3. Figure 1 demonstrates axial images for solid nodules classified as Lung‐RADS category 2 (C050), 3 (C021‐1), 4A (C012), and 4B (C261‐3) on NDCT. Figure 2 demonstrates axial images for subsolid nodules classified as Lung‐RADS category 2 (C179‐6), 3 (C128‐1), and 4A (C160‐1) on NDCT. While the CAD system successfully detected all the solid nodules on NDCT and LDCT either before or after denoising, three subsolid nodules were not identified on NDCT (C095, C179‐7, C218‐4) and four subsolid nodules were not identified on LDCT either before or after denoising (C081‐1, C179‐4, C179‐5, C218‐1). Figure 3 presents axial images of subsolid nodules that were not detected on NDCT, whereas Figure 4 shows axial images of subsolid nodules that were undetected on LDCT either before or after denoising. The mean CNR of all solid nodules was 16.65 on NDCT and 6.65 on LDCT, whereas the mean CNR of all subsolid nodules was 6.26 on NDCT and 2.47 on LDCT. Among subsolid nodules, those that were identifiable demonstrated a mean CNR of 8.38 on NDCT and 3.27 on LDCT, while unidentifiable subsolid nodules exhibited a lower mean CNR of 3.83 on NDCT and 1.56 on LDCT.

**TABLE 2:** Characteristics for patients with solid nodules.

Case	Age (years)	CTDI_vol_ (mGy)	Lung‐RADS classification
C012	47	7.32	4A ‐ Suspicious
C016	58	4	#1:3 ‐ Probably benign
			#2:2 ‐ Benign appearance
C021	85	4.71	#1:3 ‐ Probably benign
			#2:3 ‐ Probably benign
C050	17	5.84	2 ‐ Benign appearance
C077	71	6.65	4A ‐ Suspicious
C107	64	2.45	#1:3 ‐ Probably benign
			#2:3 ‐ Probably benign
C111	34	6.51	3 ‐ Probably benign
C124	48	9.04	3 ‐ Probably benign
C162	39	3.46	4A ‐ Suspicious
C170	52	6.56	3 ‐ Probably benign
C179	55	4.3	#2:2 ‐ Benign appearance
			#3:3 ‐ Probably benign
C190	30	4.15	3 ‐ Probably benign
C218	63	5.47	#3:2 ‐ Benign appearance
C227	52	10.04	2 ‐ Benign appearance
C241	55	4.77	3 ‐ Probably benign
C257	59	7.89	3 ‐ Probably benign
C258	21	10.44	4A—Suspicious
C261	58	6.76	#1:2 ‐ Benign appearance
			#2:2 ‐ Benign appearance
			#3:4B—Very suspicious
C268	34	5.84	#1:3 ‐ Probably benign
			#2:3 ‐ Probably benign

**TABLE 3:** Characteristics for patients with subsolid nodules.

Case	Age (years)	CTDI_vol_ (mGy)	Lung‐RADS classification
C081	61	4.38	2 ‐ Benign appearance^a^
C095	22	4.82	^b^
C128	76	5.89	3 ‐ Probably benign
C160	56	7.69	#1:4A ‐ Suspicious
			#2:2 ‐ Benign appearance
C179	55	4.3	#1:3 ‐ Probably benign
			#4:2 ‐ Benign appearance^a^
			#5:2 ‐ Benign appearance^a^
			#6:2 ‐ Benign appearance
			#7: ^b^
C218	63	5.47	#1:2 ‐ Benign appearance^a^
			#2:2 ‐ Benign appearance
			#4: ^b^
C232	76	7.7	2 ‐ Benign appearance
C234	72	6.57	2 ‐ Benign appearance

^a^ Lung nodule was not detected by CAD on LDCT or denoised LDCT.

^b^ Lung nodule was not detected by CAD on NDCT.

**FIGURE 5:**
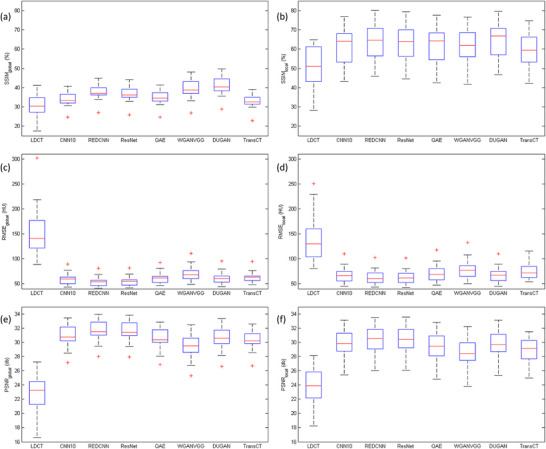
Box and whisker plots of (a) SSIM_global_, (b) SSIM_local_, (c) RMSE_global_, (d) RMSE_local_, (e) PSNR_global_, (f) PSNR_local_ between NDCT and LDCT before or after denoising for solid nodules. All data were analyzed on a per‐nodule basis.

**FIGURE 6:**
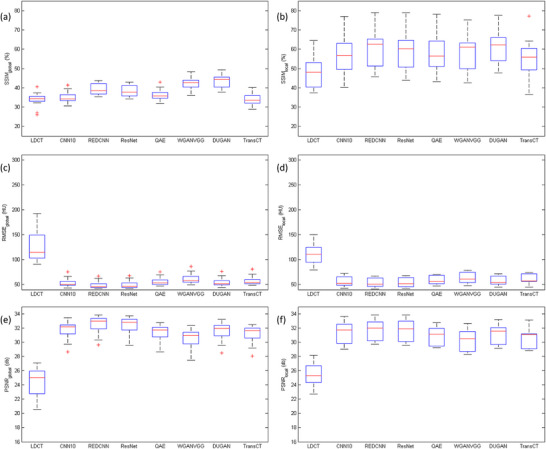
Box and whisker plots of (a) SSIM_global_, (b) SSIM_local_, (c) RMSE_global_, (d) RMSE_local_, (e) PSNR_global_, (f) PSNR_local_ between NDCT and LDCT before or after denoising for subsolid nodules. All data were analyzed on a per‐nodule basis.

Figure 5 shows the box and whisker plots of SSIM_global_, SSIM_local_, RMSE_global_, RMSE_local_, PSNR_global_ and PSNR_local_ between NDCT and LDCT before or after denoising for solid nodules. Corresponding results for subsolid nodules are shown in Figure 6. The red line in each box represents the median of the distribution, whereas the top and bottom of each box represent the 25th and 75th percentile of the distribution, respectively. The whiskers extend to the minimum and maximum values for a data set. All the data were calculated per nodule basis. Based on the paired t test, all denoising methods resulted in significant improvements in SSIM, RMSE and PSNR (*p* < 0.05). One‐way analysis of variance (ANOVA) revealed statistically significant differences among the denoised results for all global measurements (*p* < 0.05). However, for local measurements, significant difference was only observed in RMSE and PSNR for solid nodules. Among the evaluated methods, the highest SSIM_local_ was achieved by DUGAN, followed by REDCNN, ResNet, QAE, WGANVGG, CNN10, and TransCT. The lowest RMSE_local_, corresponding to the highest PSNR_local_, was observed in REDCNN, followed by ResNet, CNN10, DUGAN, QAE, TransCT, and WGANVGG.

**FIGURE 7:**
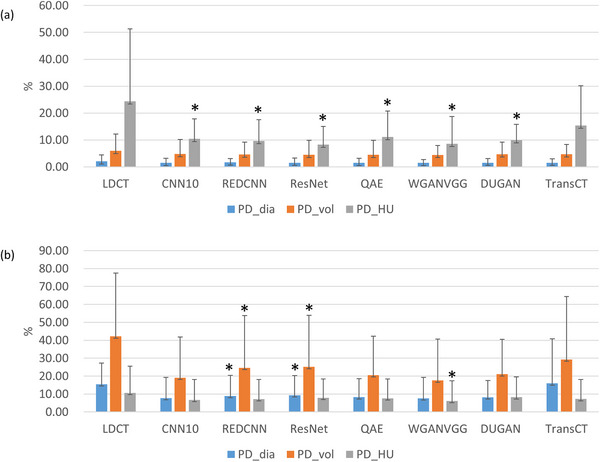
Histograms of PD_dia_, PD_vol_, PD_HU_ between NDCT and LDCT before or after denoising for (a) solid nodules and (b) subsolid nodules. All data were analyzed on a per‐nodule basis.

**FIGURE 8:**
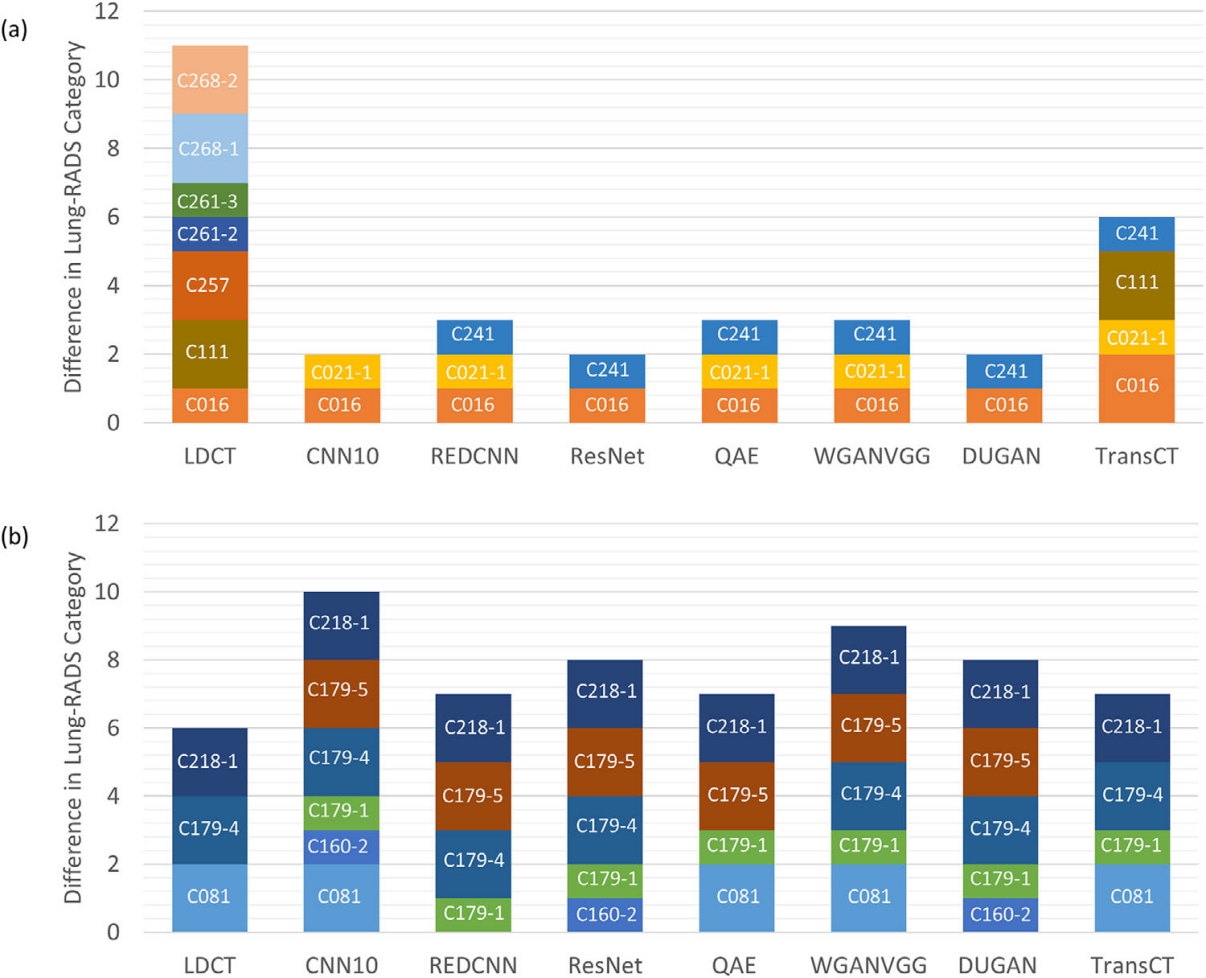
Histograms of difference in Lung‐RADS category between NDCT and LDCT before or after denoising for (a) solid nodules and (b) subsolid nodules. All data were analyzed on a per‐nodule basis.

Figure 7 shows the histograms of PD_dia_, PD_vol_, and PD_HU_ between NDCT and LDCT before or after denoising, demonstrating that image denoising improves image quality by reducing PD_dia_, PD_vol_, and PD_HU_. The asterisks in Figure 7 denote statistically significant differences identified by the paired t‐test (*p* < 0.05) between LDCT and denoised LDCT. It was found that PD_HU_ was the most sensitive metric for solid nodules, whereas the lowest PD_HU_ was found in ResNet, followed by WGANVGG, REDCNN, DUGAN, CNN10, QAE, and TransCT. As for subsolid nodules, the most sensitive metric was PD_vol_, whereas the lowest PD_vol_ was achieved by WGANVGG, followed in order by CNN10, QAE, DUGAN, REDCNN, ResNet, TransCT. Figure 8 shows the histograms of difference in Lung‐RADS category between NDCT and LDCT before or after denoising. For solid nodules, image denoising was found to improve the accuracy of Lung‐RADS categorization in LDCT, whereas misclassifications occurred most frequently with TransCT. Conversely, for subsolid nodules, none of the evaluated methods demonstrated an improvement in Lung‐RADS categorization accuracy.

## 4 DISCUSSION

This study evaluated the effects of seven deep learning‐based denoising algorithms trained within a standardized benchmark framework on LDCT images used for lung cancer screening, assessing their relative performance under consistent training and evaluation conditions. Among the evaluated methods, denoising of LDCT images resulted in a measurable improvement in overall image quality, as reflected by reduced RMSE, increased PSNR, and enhanced SSIM compared with the original LDCT. Beyond these image quality metrics, the preservation of clinically relevant nodule features was also improved after denoising. Specifically, the measurements of nodule diameter, volume, and mean HU values obtained from denoised LDCT images were more consistent with those derived from NDCT, indicating that denoising not only enhanced visual image quality but also contributed to more reliable quantitative feature extraction. These improvements translated into better accuracy in Lung‐RADS categorization for solid nodules, highlighting the potential of denoising methods to improve clinical decision‐making in this subgroup. However, such benefits were not observed for subsolid nodules, where the accuracy of Lung‐RADS classification remained limited despite denoising. As illustrated in Figure 8, CNN10, DUGAN, and ResNet achieved relatively higher categorization accuracy among the evaluated methods for solid nodules, but their performance was comparatively poorer for subsolid nodules, underscoring the continued challenges associated with accurately characterizing this nodule subtype. Image denoising can improve the detection accuracy and visual quality of lung nodules in CT images, but the denoising process inherently introduces some degree of bias into the resulting images, such as excessive oversmoothing that diminishes important structural details, or alterations in texture patterns that may distort the true appearance of tissues and potentially affect diagnostic accuracy. Zhou et al. developed a patient‐data‐based virtual imaging trial framework and applied it to assess the spatial‐resolution characteristics of a ResNet‐based deep‐CNN denoising algorithm, finding that the in‐plane and z‐axis resolution degraded under conditions of lower contrast, reduced radiation dose, and increased denoising strength.^26^ For high‐contrast objects, such as solid nodules, this bias tends to be relatively minor because their large attenuation differences produce strong signals that are less susceptible to the effects of noise. Consequently, the preservation of their quantitative features, including diameter and volume, is largely maintained, which helps explain the observed improvements in measurement consistency and Lung‐RADS categorization accuracy for solid nodules. In contrast, low‐contrast objects, such as subsolid nodules, are more vulnerable to image noise, as their smaller differences in attenuation reduce the inherent CNR.^27^, ^28^ When denoising is applied to these low‐contrast structures, the introduced bias can become more significant, potentially offsetting the benefits of noise reduction and leading to less reliable feature quantification. This difference in susceptibility to denoising‐induced bias is supported by the results presented in Figure 7. For solid nodules, the mean PD_dia_ and PD_vol_ of the evaluated methods were approximately 1.51% and 4.59%, respectively, reflecting relatively high fidelity in feature preservation. By contrast, for subsolid nodules, the mean PD_dia_ and PD_vol_ were markedly higher, at 9.42% and 22.47%, respectively, indicating substantial variability and reduced accuracy in the measurement of key nodule characteristics. These findings highlight that while denoising improves overall image quality, its impact on low‐contrast structures is more complex and can limit the clinical reliability of quantitative assessments in subsolid nodules, underscoring the need for careful evaluation of denoising methods when applied to low‐contrast lesions.

Several limitations should be considered in interpreting the results of this study. First, the dataset was acquired using Siemens CT scanners and included only 26 solid nodules and 15 subsolid nodules, which may limit the generalizability of the findings and reduce statistical power for certain comparisons. Nevertheless, an important advantage of this dataset is that it is openly accessible, thereby promoting transparency, reproducibility, and collaborative advancement in the field. Second, this study relied on a single CAD system to extract key nodule features, and no human observer assessment was incorporated for comparison. As this CAD system has been integrated into lung cancer screening programs and is routinely used in clinical practice at our institution to assist radiologists in decision‐making, understanding the potential impact of image denoising on its performance is crucial. Radiomics‐based CAD systems have gained significant attention in recent years; however, variations in input image quality remain a major factor influencing the reproducibility and reliability of radiomics analyses. Therefore, denoising LDCT scans to achieve image quality comparable to that of full‐dose scans is essential to ensure accurate lesion detection and robust radiomic feature extraction. Third, although seven deep learning‐based denoising methods were evaluated, this selection does not encompass the full spectrum of existing algorithms, and other methods may perform differently in terms of image quality improvement and preservation of clinically relevant nodule features. Despite these limitations, our findings provide insights into the potential impact of deep learning‐based denoising methods on LDCT, as quantified by objective image quality metrics and clinical implications. While objective image quality metrics, such as RMSE, PSNR, and SSIM, are useful for quantifying noise suppression and structural fidelity, they may fail to capture the clinical implications of denoising, for example, the ability to detect and characterize pulmonary nodules.^29^ Establishing standardized evaluation frameworks that link algorithmic improvements directly to diagnostic performance will be critical for translating deep learning‐based denoising into routine clinical practice, ensuring both image quality optimization and reliable clinical decision‐making.

## 5 CONCLUSION

This study evaluated the impacts of seven deep learning‐based denoising algorithms on LDCT for lung cancer screening. Our results indicated that deep learning‐based denoising significantly improved LDCT image quality, as evidenced by reduced RMSE, increased PSNR, and enhanced SSIM. In addition, denoising enhanced the preservation of nodule size and CT density, thereby aligning LDCT measurements more closely to NDCT values. These gains translated into improved accuracy of Lung‐RADS categorization for solid nodules; however, subsolid nodules remained more susceptible to both image noise and denoising‐induced biases. Incorporating denoising techniques into LDCT workflows offers considerable potential to enhance early lung cancer detection while keeping radiation exposure at a minimum. Nonetheless, validating their influence on diagnostic performance remains crucial for clinical adoption.

## AUTHOR CONTRIBUTIONS

**Shih‐Sheng Chen**: Conceptualization; methodology; software; formal analysis; writing‐review and editing. **Hsiao‐Hua Liu**: Conceptualization; methodology; software; formal analysis. **Ching‐Ching Yang**: Conceptualization; methodology; software; validation; formal analysis; writing‐review and editing; supervision.

## CONFLICT OF INTEREST STATEMENT

The authors have no competing interests to declare that are relevant to the content of this article.
